# Detecting *KRAS* mutations in peripheral blood of colorectal cancer patients by peptide nucleic acid clamp PCR

**DOI:** 10.3892/etm.2012.694

**Published:** 2012-09-03

**Authors:** SHOZO MIYANO, KISABURO HANAZAWA, TOSHIAKI KITABATAKE, MINORU FUJISAWA, KUNIAKI KOJIMA

**Affiliations:** 1 Departments of Oncology and; 2 General Surgery, Juntendo University Nerima Hospital, Tokyo 177-8521, Japan

**Keywords:** *KRAS* mutation, colorectal cancer, peptide nucleic acid, plasma

## Abstract

We investigated the effectiveness of peptide nucleic acid (PNA) clamp PCR for detecting *KRAS* mutations in peripheral blood samples of colorectal cancer (CRC) patients. We compared *KRAS* point mutations between tumour tissue and blood samples. Forty-two patients were included in this study. We observed *KRAS* mutations in formalin-fixed, paraffin-embedded tissues by PCR direct sequencing and in blood samples by PNA clamp PCR. *KRAS* point mutations were detected in primary tumour tissue samples of 13 patients (31.0%) and in peripheral blood samples of 10 patients (23.8%). *KRAS* point mutations were detected in both samples for 8 patients (19.0%). The sensitivity, specificity and accuracy for detecting *KRAS* mutations in peripheral blood and tumour tissue samples were 61.5, 93.1 and 83.3%, respectively. The positive and negative predictive values were 80.0 and 84.4%, respectively. Five patients with mutant *KRAS* in their plasma preoperatively, did not exhibit *KRAS* mutations postoperatively. Our method detected *KRAS* point mutations in peripheral blood samples of CRC patients, which contained extremely small amounts of mutant cells. This method is helpful for identifying metastatic CRC patients in whom metastases will respond to EGFR-targeted monoclonal antibody therapy.

## Introduction

Colorectal cancer (CRC) is the third most common cancer in the world (1). Extensive research has improved the survival of CRC patients, but its disease-specific mortality remains to be approximately 33% in the developed world (2). Recently, cetuximab and panitumumab, two monoclonal antibodies targeting the epidermal growth factor receptor (EGFR), have proven to be effective in combination with chemotherapy for treating CRC (3). Data from oxaliplatin and cetuximab in first-line treatment of metastatic colorectal cancer and cetuximab combined with irinotecan in first-line therapy for metastatic colorectal cancer trials have indicated that the addition of cetuximab during first-line treatment with infusional fluorouracil, leucovolin and oxaliplatin or fluorouracil, leucovolin and irinotecan was not beneficial in *KRAS*-mutated tumours, although these patients benefited from chemotherapy alone (4,5). *KRAS* point mutations are predictive markers for effective treatment with EGFR inhibitors (6).

Cetuximab and panitumumab have been approved by the US Food and Drug Administration. Both of these molecules bind to the extracellular domain of EGFR, leading to inhibition of its downstream signalling (7). EGFR is a transmembrane receptor tyrosine kinase belonging to the ErbB (also known as HER) family that is activated in many tumours. EGFR overexpression by cancer cells results in ligand-independent dimerization and activation (8). *KRAS* encodes a small G protein that links ligand-dependent receptor activation to intracellular pathways of the EGFR signalling cascade. Mutation at key sites within the gene, commonly at codons 12 and 13, causes constitutive activation of *KRAS*-associated signalling (5).

Many studies have examined new predictive factors in tumour tissue and blood samples. However, none of these markers have been introduced into clinical practice for CRC treatment, except for *KRAS* mutations as predictors of non-response to anti-EGFR treatment (9). In this study, we investigated the effectiveness of *KRAS* mutations in the blood samples of CRC patients to predict response to anti-EGFR therapy. We compared *KRAS* point mutations in tumour tissues and peripheral blood samples. Peptide nucleic acid (PNA) clamp PCR was used to detect mutations in codons 12 and 13 of the *KRAS* gene. PNA is a synthetic DNA analogue in which the ribose-phosphate backbone of the DNA is replaced by peptide bond-linked N-(2-aminoethyl)-glycine units. PNA clamp real-time PCR provides a time-sparing and sensitive method for the detection of *KRAS* point mutations using blood samples of CRC patients (10).

## Patients and methods

Forty-two eligible patients were enrolled in this study between September, 2009 and March, 2010. None of the patients had received previous therapy for CRC. A 5-ml peripheral blood sample was obtained from each CRC patient before tumour resection or endoscopic biopsy. We detected *KRAS* mutations in formalin-fixed, paraffin-embedded (FFPE) tissue samples by PCR direct sequencing and in blood samples by PNA clamp PCR. This study was approved by the Ethics Committee of Juntendo University. Written informed consent for conducting tumour tissue and blood research was obtained from all patients.

### PCR direct sequencing of FFPE samples

DNA was purified from FFPE tissue samples of CRC patients using the QIAamp DNA FFPE tissue kit (Qiagen, Venlo, The Netherlands) as described by the manufacturer’s instructions. PCR amplification of exon 2 of the *KRAS* gene was performed in a total volume of 25 μl containing 1X PCR buffer, 200 nM dNTPs, 1.5 mM MgCl*_2_*, 100 nM forward and reverse primers, 0.5 units of Platinum TaqDNA polymerase (Invitrogen, Carlsbad, CA, USA) and 25 ng of template DNA. Thermal cycling conditions were as follows: initial denaturation at 94°C for 2 min, 40 cycles at 94°C for 30 sec and 60°C for 1 min and final elongation at 72°C for 10 min. PCR products were purified by the Illustra™ GFX PCR DNA and Gel Band Purification kit (GE Healthcare, Little Chalfont, UK). DNA sequencing of PCR products was performed on a Genetic Analyzer 3130xl (Applied Biosystems, Foster City, CA, USA) using the BigDye*^®^* Terminator v1.1 Sequencing kit (Applied Biosystems) as described by the manufacturer’s instructions.

### PNA clamp PCR of plasma samples (10)

DNA was purified from 3 ml of plasma samples of CRC patients using the QIAamp Circulating Nucleic Acid kit (Qiagen) as described by the manufacturer’s instructions. PNA clamp PCR was performed in a total volume of 25 μl containing 1X Phusion HF buffer, 200 nM dNTPs, 60 nM forward and reverse primers, 500 nM PNA probe (Table I), 31.25-fold diluted SYBR-Green (1:2,000 in DMSO), 0.4 units of Phusion HF DNA polymerase (Finnzymes, Espoo, Finland and 2.5 μl of plasma DNA. Thermal cycling conditions were as follows: initial denaturation at 98°C for 30 sec, 40 cycles at 98°C for 10 sec; 76°C for 10 sec; 62°C for 20 sec and 72°C for 10 sec followed by a final cycle at 95°C for 15 sec and 60°C for 15 sec and dissociation at 95°C for 15 sec. The reaction was performed using the Sequence Detection System Prism 7900HT System (Applied Biosystems). Each sample was analysed with and without PNA. PCR products were purified by ExoSAP-IT*^®^* (USB, Cleveland, OH, USA). DNA sequencing of PCR products was performed on a Genetic Analyzer 3130xl using the BigDye Terminator v1.1 Sequencing kit as described by the manufacturer’s instructions.

## Results

We studied 42 patients with CRC, including 30 men and 12 women, with a median age of 67.5 years (range, 33–84 years) from September, 2009 to March, 2010. Patient characteristics are summarized in Table II. Twenty-nine tumours (69.0%) were present in the colon and 13 (31.0%) were present in the rectum. The primary tumours of 40 patients were resected surgically, and tumour samples of the remaining 2 patients were obtained by endoscopic biopsy. Histologically, 25 (59.5%) tumours were well-differentiated carcinomas, 16 (38.1%) were moderately differentiated carcinomas and 1 (2.4%) was a poorly differentiated carcinoma.

*KRAS* point mutations were detected in primary tumour tissue samples of 13 patients (31.0%) and in peripheral blood samples of 10 (23.8%). *KRAS* point mutations were found in both samples for 8 patients (19.0%). Primary tumour tissue samples of 10 patients showed *KRAS* point mutations in codon 12 and those of 3 patients showed mutations in codon 13. Plasma samples of 6 patients showed *KRAS* point mutations in codon 12 and those of 4 patients showed mutations in codon 13 (Table III).

The sensitivity, specificity and accuracy of the assay for detecting *KRAS* mutations in peripheral blood and tumour tissue samples were 61.5, 93.1 and 83.3%, respectively. The positive and negative predictive values were 80.0 and 84.4%, respectively (Table IV). Two patients showed *KRAS* point mutations only in their peripheral blood samples. Thirteen patients showed *KRAS* point mutations in primary tumour tissue samples, and among them 4 had a Gly12Val mutation, 3 had a Gly13Asp mutation, 3 had a Gly12Asp mutation, 2 had a Gly12Ser mutation and 1 had a Gly12Cys mutation. Ten patients showed *KRAS* point mutations in serum samples, and among them 4 had a Gly13Asp mutation, 2 had a Gly12Ser mutation, 2 had a Gly12Val mutation, 1 had a Gly12Asp mutation and 1 had a Gly12Cys mutation. Five patients showed *KRAS* point mutations only in their primary tumour tissue samples. Eight patients showed *KRAS* point mutations in both their primary tumour and blood samples, and we found a concordance of *KRAS* mutation status between blood and primary tumour tissue samples in all 8 patients (Table V). Two patients showed *KRAS* point mutations only in the blood samples, and both patients had a Gly13Asp mutation (Table VI). After 6 months, we investigated peripheral blood samples from 5 patients who had mutant *KRAS* in their plasma preoperatively. None of the patients exhibited *KRAS* mutants postoperatively (Table VII).

## Discussion

The Catalogue of Somatic Mutations in Cancer database reported *KRAS* mutations in a total of 8402 samples and no mutations in 29,328 samples (11). Point mutations were most commonly observed in codons 12 and 13 of the *KRAS* gene and less commonly in codon 61 (12). *KRAS* mutations have been found in 65–100% of pancreatic carcinomas, 36% of CRCs and 20% of non-small cell lung cancers (13). We detected *KRAS* mutations in 31.0% of the tumour tissue samples in our cases. Thus, our results are consistent with those obtained by other studies.

*KRAS* mutation analysis of primary tumour tissue samples is performed since metastatic tissue is often not available. *KRAS* genotyping in metastatic CRC patients may be prevented due to the difficulty in obtaining tumour samples from certain patients. Considering the genetic heterogeneity of CRCs, the absence of detectable *KRAS* mutations in primary tumour tissue samples does not exclude the presence of *KRAS* mutations in metastatic tissues. Whether metastatic CRC patients carry the same *KRAS* mutation as the primary tumour is controversial. Furthermore, whether *KRAS* mutations are stable or whether they undergo clonal evolution during tumour progression remains a matter of debate. Few studies have reported the presence of mutations in metastatic tissues derived from CRCs or the absence of mutations present in the primary tumour tissues in the distant recurrence (14). Klein suggested that tumour cells detach from the primary lesion before acquisition of a malignant phenotype to develop new mutations and exhibit metastatic growth at a distant site in an early dissemination model (15). The variation in mutations between primary tumours and metastases may occur, and consequently, mutations in the primary tumour may not be sufficient to predict the response of metastases to EGFR-targeted monoclonal antibodies. Some studies found a discordance of *KRAS* mutations in primary tumours and metastatic sites, with an overall discordance observed in 4–32% of patients. It is still debatable whether the assessment of *KRAS* mutations in the available primary tumour accurately indicates *KRAS* mutations in the corresponding metastasis (16).

Recently, Knijn *et al* (16) reported a concordant *KRAS* mutation status in 96.4% of 305 paired samples of colorectal tumours and liver metastases. A discordant *KRAS* status between primary tumour and metastatic tissues was observed in a small number of patients (3.6%). *KRAS* mutations are believed to emerge early during CRC progression and are associated with the growth of small adenomas to a clinically significant size (17). Therefore, *KRAS* mutations are predicted to be identical in primary tumour and metastatic tissues (15).

There is evidence that primary cancer begins shedding neoplastic cells into circulation at an early stage (18). The amount of free plasma DNA in circulation is 5- to 10-fold higher in patients with solid tumours than that in normal patients (19). In this study, we used PNA clamp real-time PCR to detect *KRAS* mutations in blood samples of CRC patients. This assay is useful for detecting mutations in codons 12 and 13 of the *KRAS* gene and is based on high-fidelity DNA polymerase. To detect a minimal amount of mutant DNA in clinical samples, PNA oligomers have been developed. PNAs are non-extendable oligonucleotides in which the ribose-phosphate backbone is replaced by peptide bond-linked 2-aminoethyl glycine units. In PNA clamp PCR, PNA oligomers suppress the amplification of the complementary sequence confined by a pair of DNA oligonucleotide primers since PNA is not a substrate for DNA polymerase (20). We believe that our method of detecting *KRAS* mutations in blood samples of metastatic CRC patients may be a good clinical tool for predicting the effectiveness of EGFR-targeted monoclonal antibodies.

Lindforss *et al* (19) reported that plasma *KRAS* mutants could be detected preoperatively and early postoperatively in all stages of colorectal neoplasia. They found that 8 of 9 patients, all of whom were radically treated, still displayed tumour-derived *KRAS* mutants in their plasma on day 3 after surgical resection. The result of our experiment was contrary to that of Lindforss *et al* (19). In our study, all 5 patients with preoperative plasma *KRAS* mutants did not show *KRAS* point mutations 6 months postoperatively.

In conclusion, our method was able to detect *KRAS* point mutations in the peripheral blood of CRC patients, which contains extremely small amounts of mutant cells. This method is helpful for identifying metastatic CRC patients in whom their metastases will respond to EGFR-targeted monoclonal antibodies. Furthermore, it is hopeful that this method will reveal micrometastases in CRC patients as tumour markers. It is likely that this method can substitute for previous methods of detecting *KRAS* point mutations in almost all CRC patients.

## Figures and Tables

**Table I:** Primers and PNA clamp probe sequences.

Name	Sequences
*KRAS*-F	5′-TGTGTGACATGTTCTAATATAGTCACATTT-3′
*KRAS*-R	5′-ATCGTCAAGGCACTCTTGCCTAC-3′
PNA clamp probe	5′-TACGCCACCAGCTCC-3′

PNA, peptide nucleic acid; F, forward; R, reverse.

**Table II:** Patient characteristics.

Characteristics	No. of patients (%)
Patients	42
Median age (range), in years	67.5 (33–84)
Gender	
Male	30 (71.4)
Female	12 (28.6)
Primary tumour site	
Colon	29 (69.0)
Rectum	13 (31.0)
Histopathological subtype	
Adenocarcinoma	37 (88.1)
Adenocarcinoma with a mucinous component	4 (9.5)
Mucinous adenocarcinoma	1 (2.4)
Differentiation	
Well	25 (59.5)
Moderately	16 (38.1)
Poorly	1 (2.4)
Depth of tumour invasion (T stage)	
T1	6 (14.3)
T2	6 (14.3)
T3	26 (61.8)
T4	2 (4.8)
Unknown	2 (4.8)
Lymph node metastases (N stage)	
N0	22 (52.4)
N1	10 (23.8)
N2	8 (19.0)
Unknown	2 (4.8)
Stage (TNM)	
0	1 (2.4)
I	10 (23.8)
IIA	10 (23.8)
IIB	3 (7.1)
IIIA	0
IIIB	6 (14.3)
IIIC	7 (16.7)
IV	5 (11.9)

**Table III:** Detection of *KRAS* mutations in tumour tissue samples and in peripheral blood samples.

	*KRAS* mutation sites		
	*KRAS* mutations n (%)	Codon 12 n (%)	Codon 13 n (%)
*KRAS* in tumor tissue samples	13 (31.0)	10 (23.8)	3 (7.1)
*KRAS* in peripheral blood samples	10 (23.8)	6 (14.3)	4 (9.5)
Blood and tissue samples	8 (19.0)	6 (14.3)	2 (4.8)

**Table IV:** Correlation between PCR sequencing and PNA clamp PCR in tumour tissue and peripheral blood.

	*KRAS* in tumor tissue		
	Positive	Negative	P-value
*KRAS* in peripheral blood			
Positive	8	2	<0.0005
Negative	5	27	

Sensitivity 61.5%, specificity 93.1%, accuracy 83.3%, positive predictive value 80.0% and negative predictive value 84.4%.

**Table V:** Eight patients with *KRAS* mutations in both the primary tumour tissue and peripheral blood samples.

Patient	Gender	Age	Mutation site	Differentiation	T	N	Stage
1	Female	71	Gly13Asp	Moderately	3	0	IIA
2	Female	81	Gly12Val	Moderately	3	0	IIA
3	Male	65	Gly12Asp	Well	4	0	IIB
4	Male	72	Gly12Ser	Moderately	4	1	IIIB
5	Female	51	Gly13Asp	Moderately	3	2	IIIC
6	Female	78	Gly12Val	Moderately	3	2	IIIC
7	Female	72	Gly12Ser	Well	3	2	IV
8	Male	74	Gly12Cys	Moderately	3	1	IV

**Table VI:** Two patients with *KRAS* point mutations in the blood samples only.

Patient	Gender	Age	Mutation site	Differentiation	T	N	M	Stage
9	Male	80	Gly13Asp	Moderately	3	0	0	IIA
10	Male	63	Gly13Asp	Moderately	3	0	0	IIA

**Table VII:** *KRAS* analysis of plasma preoperatively and 6 months postoperatively.

			Plasma *KRAS* mutation site	
Patient	Gender	Age	Preoperatively	Postoperatively
1	Female	71	Gly13Asp	Wild-type
2	Female	81	Gly12Val	Wild-type
4	Male	72	Gly12Ser	Wild-type
5	Female	51	Gly13Asp	Wild-type
6	Female	78	Gly12Val	Wild-type
